# Management of Choroidal Neovascularization Associated with Optic Disc Melanocytoma with Intravitreal Aflibercept: A Case Report and Review of the Literature

**DOI:** 10.1155/2019/2672798

**Published:** 2019-08-07

**Authors:** Hany S. Hamza, Magdy Moussa, Abdelrahman M. Elhusseiny

**Affiliations:** ^1^Department of Ophthalmology, Kasr Al-Ainy School of Medicine, Cairo University, Egypt; ^2^Department of Ophthalmology, Tanta University, Egypt; ^3^Department of Ophthalmology, Bascom Palmer Eye Institute, University of Miami Miller School of Medicine, 900 NW 17 Street, Miami, FL 33136, USA

## Abstract

**Purpose:**

To report a rare case of melanocytoma associated choroidal neovascularization (CNV) treated with intravitreal aflibercept.

**Methods:**

We reviewed the literature for the previous reports and different methods of treatment.

**Results:**

Optic disc melanocytoma has been considered as a benign melanocytic tumor that rarely affects vision. There is evidence in the literature of association between choroidal neovascular membrane and disc melanocytoma.

**Conclusions:**

In conclusion, our article provides a review of literature of such a rare association in which the ophthalmologists must be aware of its occurrence and possible ways of management.

## 1. Introduction

The optic disc melanocytoma ‘magnocellular nevus of optic disc' is a rare benign melanocytic tumor that is usually innocent but sometimes it is difficult to be differentiated from juxta-papillary choroidal melanoma [1, 2]. It usually does not affect vision. However, visual impairment may occur in some cases secondary to vascular occlusion or rarely malignant transformation [1, 2]. Association between optic disc melanocytoma and choroidal neovascularization should be considered as one of the possible causes of diminution of vision in such patients. Different treatment options have been reported to be effective in treatment of such condition. This is a report of a case of optic disc melanocytoma presented with diminution of vision secondary to choroidal neovascularization (CNV), and intravitreal aflibercept was effective in the management of the associated CNV.

## 2. Case Report

**Figure 1:**
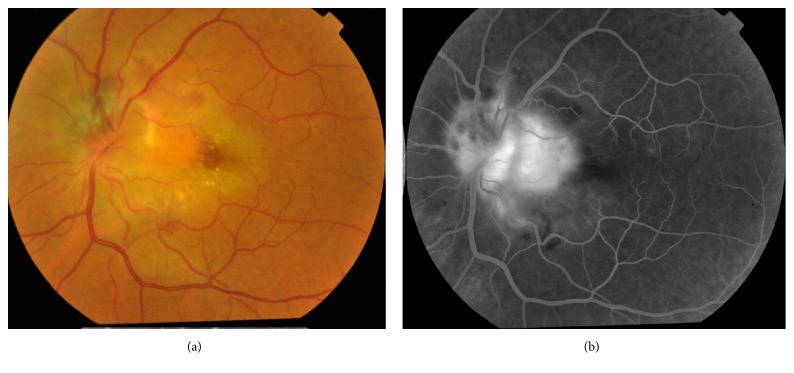
(a) Fluorescein angiography of the left eye showing early hyperfluorescence in the temporal juxtapapillary region. (b) Fundus photography of the left eye.

**Figure 2:**
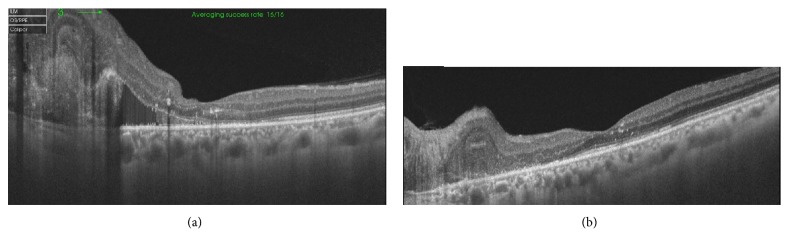
(a) Optical coherence tomography (OCT) showing diffuse macular thickening. (b) OCT showing complete resolution of the macular edema.

A 51-year-old male presented with a recent diminution of vision in his left eye of 2-month duration. The examination of the right eye was unremarkable. The ocular examination of the left eye disclosed a normal anterior segment, free ocular motility. The visual acuity of the left eye was 20/200. The fundus examination revealed a darkly pigmented mass with feathery margins on the superonasal aspect of the optic disc associated with a yellow peripapillary mass, intraretinal hemorrhages, and retinal edema adjacent to the temporal side of optic disc. Malignant transformation of melanocytoma was excluded based on absence of hemorrhage on the surface of the lesion or any signs of necrosis. Fluorescein angiography of left eye showed hypofluoresence of melanocytoma associated with early hyperfluorescence and late leakage in the temporal juxtapapillary area with deep retinal hemorrhage (Figure 1). Optical coherence tomography (OCT) showed diffuse macular thickening measuring about 800 microns with spongy edema, focal elevation of the retinal pigment epithelium temporal to the disc, and a shallow subfoveal neurosensory detachment, with absence of ‘double layer sign' (Figure 2(a)). Decision was taken to inject the patient with three intravitreal antivascular Endothelial Growth Factor (anti-VEGF) injections aflibercept (Eylea) one month apart between each of these injections. The visual acuity was improved to 20/40 using Snellen's chart. OCT was done one month after last injection showed complete resolution of macular edema (Figure 2(b)). At the 13-month follow-up period, the visual acuity is still 20/40 and dimensions of melanocytoma remained unchanged.

## 3. Discussion

Optic disc melanocytoma is a benign pigmented tumor arising from melanocytes that rarely affects vision. The most common site of melanocytoma is the optic disc; however it may rarely arise in the orbit. [10] In a minority of patients, complications may develop such as vascular occlusion, choroidal neovascularization, polypoidal choroidal vasculopathy (PCV), visual field defects, afferent pupillary defect, or rarely malignant transformation [1, 2]. Choroidal neovascularization (CNV) is a variant of wet age-related macular degeneration. The association between PCV and optic disc melanocytoma has been rarely reported, with only 2 case reports in literature [11, 12]. In our case, absence of the “double layer sign,” a typical sign of PCV on OCT, makes the diagnosis of PCV unlikely in our patient. OCT-Angiography was performed, and it did not show branching vascular network of PCV. These findings make the diagnosis of CNV more likely. However, it would have been better to do indocyanine green angiography to confirm the diagnosis and exclude PCV. CNV has been previously reported to be associated with optic disc melanocytoma with good response to intravitreal bevacizumab and ranibizumab as a method of treatment [3–8, 13]. To our knowledge, this the first case report to demonstrate the efficacy of aflibercept in the management of choroidal neovascularization associated with optic disc melanocytoma.

There are several methods of management choroidal neovascular membrane associated with optic disc melanocytoma including photodynamic therapy, submacular surgery, and intravitreal anti-VEGF injection [3, 4, 9] (Table 1).

Less than 1% of cases of optic disc melanocytoma have been associated with CNV [13]. Although the relation between both signs is unclear, we agree with Kamisasaku et al. who postulated that the tumor may produce local inflammatory mediators disrupting the function of Bruch's membrane stimulating the choroid to produce a neovascular complex [5].

Intravitreal aflibercept is effective in treatment of CNV associated with disc melanocytoma.

## Figures and Tables

**Table 1:** Review of previous reports on melanocytoma associated choroidal neovascularization (CNV).

Case	Chalam [3], 2006	Tran [4], 2006	Kamisasaku [5], 2012	Al-Halafi [6], 2013	Batmanabane et al. [7]	Urrets-Zavalia [8]	Rodrigues [9]	Current case
Age	Middle age	45 years	63 years	45 years	28 years	50 years	49 years	51 years

Gender	Male	-	Male	Male	Female	Male	Female	Male

Affected eye	Right	left	Left	Right	Left	Right	Left	Left

Primary presentation	Diminution of vision	Diminution of vision	Diminution of vision	Diminution of vision	Diminution of vision	Diminution of vision	Diminution of vision	Diminution of vision

Visual Acuity at 1^st^ presentation	20/200	20/100	0.9 LogMAR	20/160	20/40	20/80	Counting fingers 50 cm	20/200

Location of choroidal neovascularization	Juxtafoveal	Peripapilla y	Adjacent to optic disc	Juxtafoveal	Peripapillary extending to the macula	Peripapillary	Macular	Peripapillary

Treatment	Photodynamic therapy	Vitrectomy and submacular surgery	Intravitreal Bevacizumab (3 injections)	Intravitreal Bevacizumab (1 injection)	Intravitreal Ranibizumab (1 injection)	Intravitreal Bevacizumab	Intravitreal anti-VEGF was recommended	Intravitreal Aflibercept (3 injections)

Follow-up	6 months	14 months	12 months	12 months	6 months	36 months	Lost to follow-	13 months
